# Conventional Chromatographic Techniques and Biosensors for Mercury Speciation in Seafood: A Systematic Review

**DOI:** 10.3390/foods15060971

**Published:** 2026-03-10

**Authors:** Doaa Abouelenein, Miguel Henares, Ana Fuentes, Isabel Fernández-Segovia, José M. Barat, Katrin Loeschner, Lene Duedahl-Olesen, Maribel Gómez-Gómez, Amadeu Griol, Jens J. Sloth

**Affiliations:** 1 Research Group for Analytical Food Chemistry, National Food Institute, Technical University of Denmark (DTU), Henrik Dams Allé B202, DK-2800 Lyngby, Denmark; ddaab@food.dtu.dk (D.A.); kals@food.dtu.dk (K.L.); lduo@food.dtu.dk (L.D.-O.); 2 University Institute of Food Engineering–FoodUPV, Universitat Politècnica de València, Camino de Vera s/n, 46022 Valencia, Spain; mhenoli@alumni.upv.es (M.H.); anfuelo@upvnet.upv.es (A.F.); isferse1@tal.upv.es (I.F.-S.); jmbarat@tal.upv.es (J.M.B.); 3 Nanophotonics Technology Center, Universitat Politècnica de València, Camino de Vera s/n, 46022 Valencia, Spain; migmegme@ntc.upv.es (M.G.-G.); agriol@upvnet.upv.es (A.G.)

**Keywords:** mercury species, methylmercury, hyphenated techniques, food safety, analytical methods, biosensors

## Abstract

Mercury (Hg) is of significant concern due to its toxicity, which strongly depends on its chemical forms, and organic mercury compounds, particularly methylmercury (MeHg), are considered the most toxic species. Therefore, mercury speciation analysis is essential for accurate exposure and risk assessment. The primary dietary source of mercury exposure for humans is food consumption, particularly seafood. Consequently, numerous studies have focused on developing analytical techniques for the identification, characterization, and quantification of Hg species in seafood. This review evaluates and compares recent developments (2014–2025) in analytical techniques for the identification and quantification of Hg species in seafood, focusing on both traditional chromatographic methods and emerging methodologies based on biosensors. Hyphenated techniques such as HPLC–ICP-MS and GC–ICP-MS have enabled significant advancements in mercury speciation analysis. Although chromatographic methods are highly effective and widely accepted due to their accuracy and sensitivity, they often require costly instrumentation, skilled operators, and lengthy analysis times. Biosensors are increasingly proposed as alternatives; however, their applicability to seafood analysis remains limited despite advantages such as portability, simplicity, and rapid response. They are still under development and face challenges in selectivity, stability, and standardization. This review provides an overview of existing methodologies, comparing their advantages and limitations, aiming to guide improvements toward optimal methods incorporating all advantageous features.

## 1. Introduction

### 1.1. Mercury Exposure and Toxicity: A Public Health Concern

Mercury is a naturally occurring element in the air, water, and soil and can have serious health effects on humans. The most well-known forms include elemental mercury (Hg^0^), inorganic mercury (iHg), methylmercury (MeHg) and ethylmercury (EtHg). Mercury can be released into the environment through natural processes (such as volcanic activity or weathering of mercury-containing rocks) and human activities (such as metal production and cement manufacturing) [1]. The main source of mercury exposure is food consumption, especially fish and seafood. Plant foods may also be contaminated due to the use of phosphate fertilizers, which may contain appreciable amounts of toxic elements such as mercury, cadmium, and lead [2].

The toxicity and environmental impact of Hg depend significantly on its chemical form rather than its total concentration, with organic mercury compounds, particularly MeHg, being the most toxic [1,2]. MeHg is one of the most bioaccumulated trace metal species in the marine environment. Its presence in food, especially seafood, remains a big concern. Following ingestion, MeHg is efficiently absorbed and distributed systemically, with well-established neurotoxic effects. The central nervous system, especially during fetal development, is the main target for organic mercury exposure. Toxic effects include sensory alterations (sight and hearing), impaired motor coordination, memory issues, attention deficits, and learning difficulties. Meanwhile, iHg exposure leads to renal lesions, neurotoxicity, and cardiovascular disorders. In extreme cases, mercury poisoning can result in death, as seen in historical incidents such as Minamata disease (Japan) and Niigata Minamata disease, where industrial mercury discharges led to widespread poisoning resulting in severe health issues and numerous deaths among populations [3]. Due to its high toxicity, maximum levels for THg (expressed as Hg) in fish and seafood are established under European Union legislation. According to Commission Regulation (EU) 2023/915, the maximum level for total mercury in fish muscle meat is set at 0.3 or 0.5 mg/kg depending on fish species, with a higher limit of 1.0 mg/kg applied to specific predatory species such as shark, swordfish, tuna, and pike [4]. In addition, the European Food Safety Authority (EFSA) established a tolerable weekly intake (TWI) for methylmercury of 1.3 µg/kg body weight, expressed as mercury, based on developmental neurotoxicity as the critical endpoint [5,6].

### 1.2. Importance of Mercury Speciation in Seafood Safety

Most available Hg data in seafood is typically based on total mercury (THg) content. When evaluating MeHg exposure, it is often assumed that MeHg constitutes a large fraction of THg in fish muscle (commonly reported around 80–90%); however, this proportion may vary substantially across species, tissues, and environmental conditions [3]. Some studies challenge this hypothesis by suggesting that MeHg may constitute a smaller proportion of THg in certain types of seafood such as mussels, catfish, and tilapia [7]. Similarly, recent studies have shown that MeHg does not account for the majority of THg even in all predatory fish species. Recently, species-specific Hg speciation patterns were reported in billfishes, with blue and black marlins exhibiting unusually low MeHg proportions (≈10–15%) despite high THg levels [8]. Consequently, reliance on THg measurements introduces uncertainty into exposure assessment and regulatory decision making, particularly for seafood species with variable MeHg fractions.

### 1.3. Analytical Approaches for Mercury Speciation in Seafood

Typical methods for the determination of THg content rely on sophisticated instruments, such as inductively coupled plasma mass spectrometry (ICP-MS), atomic fluorescence spectroscopy (AFS), and direct mercury analyzers based on thermal decomposition–amalgamation atomic absorption spectrometry. These methods are highly sensitive; however, when used alone they can only determine THg contents. Consequently, for mercury speciation, chromatographic separation techniques coupled with atomic spectrometric detectors are required. Mercury speciation involves three steps: sample preparation, separation and detection. Hg species can be extracted from matrices by various techniques, then analytes are separated using different separation modes depending on their chemical and physical properties (polarity, solubility, ionic charge, and molecular mass). After separation, the procedure requires a mercury-specific detector to determine the concentration of each Hg species.

Several reviews have previously addressed mercury speciation in environmental and biological matrices; however, they do not focus on seafood-specific analytical constraints [9,10,11]. Yang et al. [12] highlight recent progress (from 1990 to 2020) in the field of mercury speciation using non-chromatographic atomic spectrometry in different matrices (fish, water, oil, blood, hair, etc.). Favilli et al. [1] summarized and critically examined different approaches for Hg species quantification in food, environmental and biological samples exclusively using HPLC-ICP-MS. Nonetheless, no recent review has provided a matrix-specific and side-by-side evaluation of chromatographic and biosensor-based approaches dedicated exclusively to seafood, with emphasis on analytical performance, extraction-related techniques, and practical applicability for food control. To address this gap, the present review provides a comprehensive overview of the techniques employed for the extraction and chromatographic analysis of Hg species in seafood, emphasizing key analytical parameters such as limits of detection (LODs), linearity ranges, and recoveries, as reported in studies published from 2014 to 2025.

In parallel with advancements in chromatographic techniques, the past decade has witnessed the emergence of biosensors as promising alternatives for mercury speciation. Their portability, low cost, and user-friendly operation make them attractive for on-site and rapid analysis—particularly relevant for seafood safety monitoring. Despite these advantages, the use of biosensor detectors for Hg species analysis in seafood has not been thoroughly addressed in existing review papers, as most biosensor studies focus on aqueous samples or single Hg species, limiting direct comparison with chromatographic reference methods used in food control laboratories. Therefore, this review summarizes current chromatographic methods for mercury speciation in seafood and explores recent developments in biosensing technologies applied to the same matrices. By directly comparing the analytical performance of both approaches, this work provides a unique, matrix-specific evaluation of traditional versus emerging detection strategies. Practical aspects such as methodology, LODs, and analysis time are critically assessed.

## 2. Methods

### 2.1. Literature Search

This review was conducted in accordance with the Preferred Reporting Items for Systematic Reviews and Meta-Analyses (PRISMA) 2020 statement [13]. The completed PRISMA checklist is provided as Supplementary Material. A systematic literature search was performed using three bibliographic databases: PubMed, Web of Science Core Collection, and Scopus. The search covered publications from January 2014 to December 2025 and aimed to identify studies reporting analytical methods for mercury speciation in seafood and marine food matrices.

The search strategy was designed to capture studies related to mercury speciation, analytical techniques, and seafood matrices. Keywords related to mercury speciation included “*mercury speciation*”, *methylmercury*, *MeHg*, and “*mercury species*”. Analytical-technique-related terms encompassed chromatographic methods and detection approaches, including *chromatograph*, *HPLC*, *LC*, *GC*, “*liquid chromatography*”, “*gas chromatography*”, “*capillary electrophoresis*”, “*ICP-MS*”, “*AFS*”, and “*AAS*”, as well as sensor-based methodologies such as “*biosensor*”, “*sensor*”, “*electrochemical*”, “*colorimetric*”, and “*fluorescent*”. Seafood-related terms included “*seafood*”, “*fish*”, “*shellfish*”, “*crustacean*”, “*mollusk*”, “*shrimp*”, “*tuna*”, “*salmon*”, “*seaweed*”, and “*marine*”.

Searches were conducted using the standard search fields of each database (PubMed: *All Fields*; Web of Science Core Collection: *Topic*; Scopus: *Title*, *Abstract*, *and Keywords*). All retrieved records were exported to Mendeley Reference Manager for duplicate removal and subsequent screening.

### 2.2. Study Selection Criteria

Studies were included in the review if they met the following criteria:

(i) mercury speciation analysis was performed in fish or seafood products;
(ii) the analytical approach was based on chromatographic techniques (including HPLC and non-HPLC methods) or biosensor-based methods; and
(iii) the study was a peer-reviewed original research article published in English.

The scope of the review was restricted to studies within the fields of food science and technology, analytical chemistry, applied chemistry, biochemistry, biotechnology, and nutrition. Studies were excluded if they met any of the following criteria: non-peer-reviewed documents (e.g., conference proceedings, theses, book chapters), review articles, studies not focused on seafood or marine food matrices, investigations reporting total mercury only without speciation, or studies in which the analytical methodology was not sufficiently described to allow critical evaluation.

### 2.3. Selection Process of the Articles

Duplicate records were removed prior to screening. Titles and abstracts were independently screened by two reviewers based on the predefined inclusion and exclusion criteria. Discrepancies were resolved through discussion until consensus was reached. Full-text articles of the remaining records were subsequently assessed for eligibility following the same procedure. As this review focused on analytical methodology and performance characteristics, no formal risk-of-bias tool was applied; instead, methodological quality was evaluated based on the clarity of analytical procedures, validation parameters, and completeness of reporting.

For the studies included in the final review, the following information was extracted and evaluated: analytical technique, food matrix, sample preparation and extraction procedures, method validation parameters, and reported concentration ranges. The selected studies were classified according to the analytical approach employed, including chromatographic methods and biosensor-based techniques.

## 3. Results

### 3.1. Results of the Bibliographic Search

**Figure 1:**
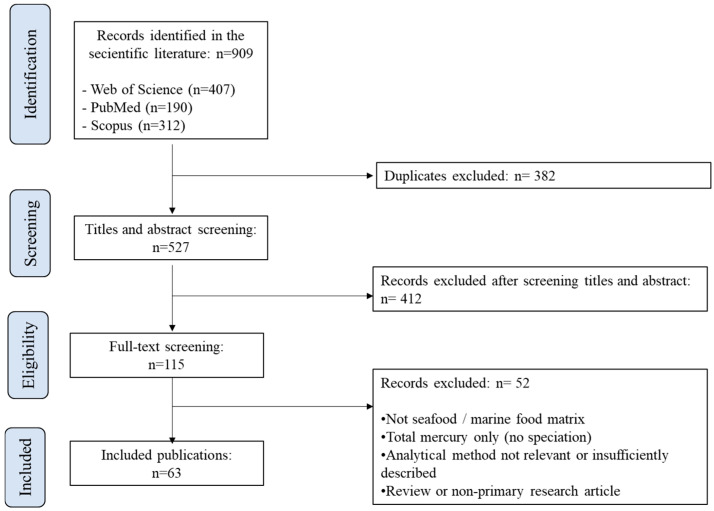
PRISMA Flow Diagram for search and screening processes.

As illustrated in the PRISMA flow diagram (Figure 1), a total of 909 records were identified through database searching in PubMed, Web of Science Core Collection, and Scopus for the period from 2014 to 2025. After removal of 382 duplicate records, 527 records remained for title and abstract screening. During this screening stage, 412 records were excluded based on lack of relevance to the review scope. As a result, 115 full-text articles were assessed for eligibility.

Following full-text evaluation, 52 articles were excluded for reasons including absence of mercury speciation analysis (e.g., total mercury only), non-seafood or non-marine food matrices, insufficient description of the analytical methodology, or classification as review or non-primary research articles. Ultimately, 63 studies met all inclusion criteria and were included in the final review. The included studies were classified according to the analytical approach employed, comprising chromatographic techniques (e.g., HPLC, GC, and related methods) and biosensor-based approaches, including colorimetric, electrochemical, and fluorescence-based sensors.

### 3.2. Sample Preparation for the Mercury Speciation in Seafood Samples Prior to Chromatographic Analysis

Sample preparation emerges as the most critical determinant of analytical reliability in mercury speciation [9] in which mercury’s volatility, affinity for surfaces, and susceptibility to redox and methylation reactions make it particularly vulnerable to artefacts introduced during extraction and pretreatment [1,9]. Across the chromatographic studies summarized in Table 1, most studies rely on acidic extraction, typically using hydrochloric (HCl) or acetic (AcOH) acids, often in combination with sulfur-containing stabilizing agents. This approach dominates routine mercury speciation, particularly for fish muscle and other lean seafood matrices, owing to its operational simplicity, compatibility with aqueous chromatographic systems, and generally reliable extraction performance. The frequent inclusion of sulfur-containing stabilizers—such as L-cysteine or 2-mercaptoethanol (2-ME)—significantly improves analytical performance. These ligands stabilize Hg species through strong Hg–S interactions, reduce adsorption losses on container walls and chromatographic surfaces, and suppress species interconversion during extraction and analysis. Alkaline digestion using potassium hydroxide (KOH) or tetramethylammonium hydroxide (TMAH) is less common. Studies applying alkaline digestion predominantly target lipid-rich or structurally complex matrices, such as fish liver, fish oil, whale tissue, and composite biological samples. Enzymatic digestion using proteolytic enzymes such as trypsin, protease XIV, or lipase appears less frequently than acidic or alkaline extraction approaches, even though it offers a chemically mild approach for matrix degradation. Its limited use is mainly related to practical issues, such as longer processing times, enzyme specificity, and the lack of thorough validation across a wide range of seafood matrices.

**Figure 2:**
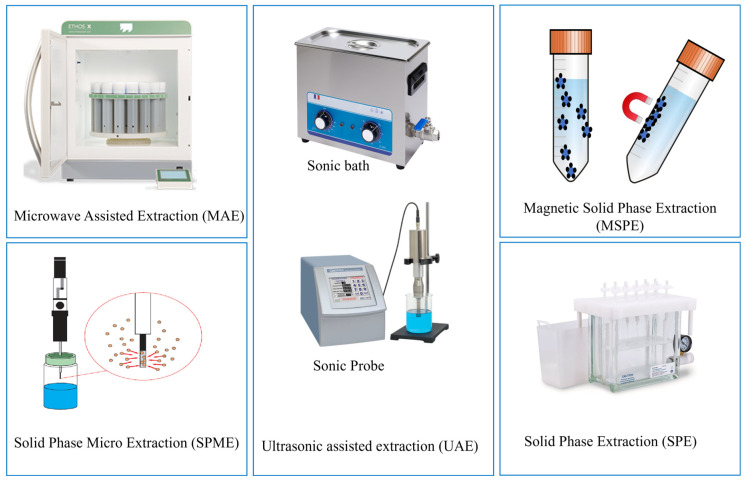
Extraction methods for seafood samples prior to chromatographic analysis (created with BioRender.com).

In addition to the choice of chemical extraction environment, the reviewed literature shows a clear tendency to employ assisted and integrated extraction techniques to improve efficiency, reproducibility, and control over species stability. Among these, ultrasound-assisted extraction (UAE) and microwave-assisted extraction (MAE) emerge as the most commonly adopted approaches, reflecting their ability to enhance matrix disruption while maintaining relatively mild chemical conditions. In addition to assisted extraction, sorbent-based strategies, including solid phase extraction (SPE) and magnetic solid phase extraction (MSPE), are often incorporated into mercury speciation workflows as downstream clean-up or enrichment steps. The main extraction strategies used prior to chromatographic analysis are summarized in Figure 2, while their respective advantages and limitations are compared in Table 2.

#### 3.2.1. Microwave-Assisted Extraction (MAE)

MAE has emerged as an effective strategy for mercury speciation in seafood, particularly for matrices characterized by strong mercury–protein or mercury–lipid interactions. By providing rapid and uniform volumetric heating, MAE accelerates tissue disruption and promotes efficient release of mercury species under controlled conditions. Nevertheless, extraction efficiency and species stability are highly dependent on operational parameters, including reagent composition, temperature, and irradiation time, which together govern the balance between matrix disruption and chemical preservation of mercury species [2]. A previous study evaluated eight different methods for extracting Hg species from tuna fish certified reference material (BCR-464). These included alkaline extraction using KOH or TMAH, acid leaching with hydrochloric, nitric, or acetic acid, a method involving L-cysteine·HCl in a 60 °C water bath, and enzymatic digestion using protease XIV (hybridization). Among these, alkaline digestion combined with MAE or UAE proved to be the most efficient, yielding the highest recoveries and the lowest levels of Hg species transformation [65].

The sensitivity of MAE to extraction conditions is further highlighted by studies focusing on MeHg stability under microwave irradiation. Chen et al. [66] developed an MAE–GC-MS method for MeHg determination using an alkaline TMAH solution and demonstrated that careful optimization of digestion conditions was critical. Their study showed that higher irradiation power or longer heating times caused a decrease in MeHg levels, likely due to thermal degradation.

Comparative studies consistently show that acidic MAE conditions increase the risk of species transformation and reduced recovery compared to alkaline extraction. Skip., It was reported that MAE using concentrated nitric or acetic acid resulted in the lowest extraction efficiencies and increased interconversion between mercury species. To mitigate these effects, several studies have employed thiol-containing extractants under acidic conditions [65]. Koplík et al. [14] used 2-ME acid solution to extract MeHg and iHg from fish, vegetables, herbs, and grains followed by LC-ICP-MS analysis. Additionally, Döker et al. [20] employed dilute HCl and 2-ME as an extractant for Hg species from commercialized fish samples, achieving reliable determination when coupled with RP-LC and ICP-MS detection.

Besides chemical digestion, enzymatic approaches have also been incorporated into MAE-based workflows. Ribeiro et al. [59] developed an MAE technique with broad applicability. The procedure achieved extraction efficiencies over 90% for a variety of seafood samples, including fish, shellfish, and seaweed. While enzymatic digestion offers a chemically mild alternative that can reduce species transformation, its broader adoption remains limited by longer processing times and the lack of harmonized protocols.

Taken together, the literature indicates that MAE performance is influenced more by how extraction conditions are managed than by the specific choice of acidic or alkaline media. When parameters such as reagent composition, temperature, and energy input are carefully controlled, MAE can be applied effectively across a range of seafood matrices. At the same time, the limited harmonization of extraction conditions continues to make direct comparison between studies more challenging.

#### 3.2.2. Ultrasound-Assisted Extraction (UAE)

UAE has been applied as a simpler alternative to MAE for mercury speciation, particularly in laboratories lacking access to specialized digestion systems. Its milder operational conditions and lower cost make it attractive for routine applications, especially in laboratories lacking access to microwave systems [67]. Sonication time, power, acid content, and sample particle size are important variables influencing UAE efficiency. However, the mechanical energy and acidic conditions commonly employed in UAE may also influence Hg species stability, increasing the risk of partial interconversion when extraction parameters are not carefully controlled.

The type of ultrasonic device used has a measurable impact on method performance. Although ultrasonic probes may improve extraction efficiency, they can increase variability and species alteration compared to ultrasonic bath systems [46,49,58]. Chen et al. (2015) [21] used an ultrasonic bath with concentrated HCl prior to HPLC–ICP–MS analysis and achieved high MeHg recoveries in pelagic fish samples. Similarly, Suratno et al. [58] applied ultrasonic bath extraction using a thiol-stabilized acidic medium to extract MeHg from fish protein certified reference material (DORM-4) and shark meat, achieving reliable results when coupled to HPLC–ICP–MS.

More complex UAE configurations have also been explored. López et al. [68] compared conventional acid leaching with enzymatic hydrolysis assisted by ultrasonic probe sonication and reported high recoveries for both approaches, highlighting the potential of UAE to support chemically mild extraction strategies. Hybrid systems combining ultrasonic probes and ultrasonic baths have been shown to further enhance extraction efficiency, increasing organic mercury extraction by approximately 40% compared to individual systems [69]. Although UAE offers operational simplicity and reduced costs, its strong dependence on sonication parameters and extractant composition limits method robustness, and the potential for Hg species transformation remains insufficiently addressed in many reported applications.

#### 3.2.3. Solid Phase Extraction (SPE)

SPE is frequently employed as a clean-up and preconcentration step in mercury speciation workflows [1]. However, strong interactions between mercury species and functionalized sorbents may also lead to selective retention or incomplete elution, with the potential to distort species distribution and bias speciation results.

To address these challenges, a range of advanced SPE strategies have been developed, largely through the functionalization of sorbent materials. Among these, magnetic solid phase extraction (MSPE) has attracted particular attention. In MSPE, functionalized magnetic sorbents are dispersed directly into the sample matrix to facilitate interaction between Hg species and selective surface ligands. Once bound, the analytes are separated from the matrix by magnetic collection and subsequently eluted for analysis [70]. While this approach simplifies phase separation, repeated adsorption–desorption cycles may affect species stability if binding affinities differ among inorganic and organic mercury forms.

Functionalized magnetic sorbents used in MSPE have demonstrated efficient extraction and preconcentration of Hg species from seafood and environmental matrices, including γ-mercaptopropyltrimethoxysilane-modified Fe_3_O_4_@SiO_2_, dithizone functionalized magnetite-reduced graphene oxide, and sulfur-functionalized magnetic polymers. Nevertheless, differences in sorbent affinity toward individual mercury species may result in selective retention or incomplete elution, limiting their applicability for comprehensive speciation [26,43,53].

In addition to magnetic strategies, smart polymeric materials known as ion-imprinted polymers (IIPs) have been explored for their high selectivity. These materials are synthesized by incorporating mercury ions as templates during polymerization, creating specific recognition cavities in the polymer matrix. Once the template is removed, the resulting IIP selectively rebinds mercury ions from complex samples [71]. A notable example is the method optimized by Jinadasa et al. [49], who reported the development of an IIP using a ternary polymerization system, achieving very low detection limits for both methylmercury and inorganic mercury in fish samples. While such selectivity is advantageous for targeted determination, it may restrict broader speciation when multiple mercury species coexist in complex seafood matrices.

Carbon nanotube (CNT)-based sorbents offer high sorption capacity but may suffer from non-selective binding. Deng et al. [22] developed a matrix solid phase dispersion (MSPD) method utilizing multi-walled CNTs as sorbents, which enabled online coupling with HPLC-ICP-MS. This setup enhanced sample dispersion, improved eluent diffusion, and achieved low LODs for iHg and MeHg, demonstrating the potential of CNT-based materials in rapid and efficient Hg species extraction. Recently a QuEChERS-inspired SPE–GC–MS method for MeHg determination in fish and shellfish was used to replace conventional liquid–liquid extraction with hydrophilic–lipophilic balance SPE. This approach eliminated hazardous solvents, reduced emulsion formation, and achieved satisfactory recoveries, demonstrating potential for routine seafood analysis [63]. Overall, SPE-based strategies provide effective clean-up and enrichment, but their impact on species stability remains sorbent-dependent and insufficiently standardized.

#### 3.2.4. Solid Phase Microextraction (SPME)

SPME is a solvent-free sample preparation technique that enables simultaneous extraction and preconcentration of analytes prior to chromatographic analysis [72]. When used for mercury speciation, SPME is most commonly applied in combination with gas chromatography, where analytes can be thermally desorbed directly from the fiber into the GC inlet. Effective application generally requires prior chemical derivatization to convert mercury species into volatile forms suitable for GC separation and detection. While this integration of extraction and transfer offers clear advantages in terms of solvent reduction and workflow simplification, the mandatory derivatization step represents a major limitation. Derivatization reactions may introduce artefacts or alter the original species distribution, particularly if reaction conditions are not rigorously controlled. In addition, the performance of SPME is strongly influenced by fiber coating chemistry, extraction equilibrium, and matrix composition.

Several applications have demonstrated the successful use of SPME in mercury speciation, especially when targeting organic Hg species in seafood and environmental samples. Lin et al. [25] applied headspace SPME using porous carbon fibers prior to GC–DBD–OES analysis following derivatization with sodium tetraphenylborate. Their results highlighted strong fiber-dependent selectivity, with no single coating enabling efficient simultaneous extraction of all mercury species. These findings underscore both the analytical potential and the inherent limitations of SPME-based workflows.

As summarized across the studies listed in Table 1, mercury speciation in seafood remains characterized by highly variable extraction conditions, limits of detection, and recoveries, highlighting the absence of harmonized analytical protocols.

### 3.3. Conventional Chromatographic Techniques

Hyphenated techniques are the most commonly employed for mercury speciation in seafood, combining highly effective chromatographic separation techniques (GC, HPLC and CE) with highly sensitive element-specific detection techniques (ICP-MS, AFS and AAS). Chromatographic separation remains the reference approach for mercury speciation in seafood due to its ability to resolve multiple species prior to element-specific detection. Among available detection strategies, ICP-MS-based hyphenated techniques dominate mercury speciation in seafood due to their sensitivity, selectivity, and compatibility with isotope dilution, such as: HPLC-ICP-MS [26,52,73], IC-ICP-MS [33,74], GC-ICP-MS [23,25,75] and CE-ICP-MS [34]. Among them, HPLC–ICP-MS is the most frequently applied platform, whereas CE–ICP-MS is less commonly used due to sensitivity and repeatability constraints. Key advantages and limitations of LC, GC, and CE for mercury speciation are summarized in Table 3.

The choice of chromatographic separation technique directly influences method sensitivity, robustness, and susceptibility to matrix effects.

#### 3.3.1. Liquid Chromatography (LC) and Related Separation Approaches

Recent LC-based approaches for mercury speciation in seafood are summarized in Table 1. The most widely used approach is reversed phase liquid chromatography (RP-LC), which separates molecules based on interactions between analytes in the polar mobile phase and lipophilic ligands attached to the column’s stationary phase. Usually, the stationary phases are made of siloxanes bound to hydrophobic substituent groups containing eighteen (C18) [49,58] or eight (C8) [16,19,29] carbon atoms. To reduce analytical costs and improve accessibility, alternative LC approaches have been developed using ion chromatography coupled to ICP-MS. In particular, anion- [45,55,60] and cation [57,59] exchange columns have been explored for mercury speciation in seafood. While these approaches offer rapid and cost-effective separation, they may exhibit poorer resolution between closely related species, such as MeHg and EtHg or MeHg and iHg, compared with RP columns. Within this context, Spanu et al. [31] proposed a simple, low-cost method using a strong anionic exchanger to block iHg during MeHg determination in fish. Similarly, Li et al. [33] used two cation guard columns to separate three Hg species in seafood, achieving LODs of 0.02–0.05 μg/L.

Mobile phase composition plays a central role in achieving efficient separation and stable detection. Most LC methods employ aqueous mobile phases containing buffers (e.g., NH_4_Ac), organic modifiers (e.g., MeOH), ion pairs, or chelating agents (e.g., L-cysteine and 2-ME) in different combinations. L-cysteine and 2-ME are frequently employed as thiol ligands for Hg, enabling efficient species separation while minimizing memory effects. Both thiol ligands are often used in combination, as complete separation using L-cysteine alone is frequently insufficient, whereas exclusive use of 2-ME can lead to excessive retention times [9]. NH_4_Ac serves as a buffer to regulate pH, and MeOH with concentrations up to 4–5% enhances mercury detection sensitivity and reduces the retention time. Higher MeOH concentrations, however, are known to suppress ICP-MS sensitivity due to reduced plasma ionization efficiency. It also provides suitable elution strength for the hydrophobic 2-ME or cysteine complexes of Hg species [1]. Some authors also reported the use of L-cysteine alone [18] or together with L-cysteine·HCl·H_2_O [76] at low concentrations as the mobile phase for mercury speciation in seafood.

Thin-layer chromatography (TLC) has also been reported for Hg separation in seafood [77]. In this technique analytes react with a solution of complexing agents (0.02% dithizone solution in chloroform) to improve separation in TLC using a silica gel plate.

#### 3.3.2. Gas Chromatography (GC)

GC-based techniques coupled to MS, AFS, or ICP-MS detectors can achieve analytical performance comparable to LC-based methods. However, a defining limitation of GC approaches is the requirement for derivatization of ionic mercury species prior to analysis. To enable GC separation, Hg species must be converted into non-polar, thermally stable dialkyl derivatives that are compatible with GC columns.

Conventional derivatization methods using Grignard reagents require anhydrous conditions, necessitating liquid–liquid extraction into a non-polar solvent followed by desiccation, making sample preparation laborious and time-consuming [78,79]. For that reason, GC-based mercury speciation has largely shifted toward alkylation using tetraalkylborate reagents, enabling derivatization directly in aqueous media. This approach significantly simplifies workflows, shortens analysis time, and reduces solvent consumption, representing a clear advantage over Grignard-based methods [79].

Among tetraalkylborates, sodium tetraethylborate (NaBEt_4_) is the most widely used, as it simplifies derivatization procedures [51,54,56]. Although NaBEt_4_ solutions are relatively stable, fresh preparation is generally recommended. A notable limitation of direct aqueous ethylation is the inability to distinguish ethylmercury from inorganic mercury species, as well as the potential formation of artefactual MeHg due to methyl impurities in the reagent—particularly problematic in samples with high iHg concentrations [78,79]. Consequently, NaBPr_4_ and NaBPh_4_ were also proposed as alternative derivatizing agents [23,25]. Both are commercially available and more stable than NaBEt_4_ when used for mercury speciation [78].

Typically, GC-based mercury speciation workflows begin with alkaline digestion using KOH or TMAH, followed by liquid–liquid extraction to extract the Hg species from the sample using a suitable organic solvent (chloroform or acetone). In some cases, a back-extraction step may be employed to transfer Hg species from the organic solvent back into aqueous solution for further processing. This step may involve the addition of a suitable aqueous solution (e.g., cysteine aqueous solution) and pH adjustment (using phosphate or sodium acetate buffers). Finally, Hg species are derivatized, forming volatile species, and the excess solvent used in the extraction or derivatization steps is removed.

A comparison between GC-MS, GC-ICP-MS, and GC-pyro-AFS for Hg speciation analysis in different matrices reported the robustness of all methods [80]. The three hyphenated techniques were precise (RSD < 5%) and sensitive (with LODs 0.05–0.21 pg for GC-ICP-MS, 1–4 pg for GC-MS, and 2–6 pg for GC-pyro-AFS). However, all systems are sufficiently sensitive, with GC–MS and GC–ICP-MS offering isotope analysis capabilities for the use of SSID and GC-pyro-AFS being the most cost-effective alternative [80]. Further method validation using GC–MS reported LODs of 1.6 and 1.9 μg/kg and LOQs of 4.6 and 5.9 μg/kg for MeHg and EtHg, respectively [23]. Recently, Wu et al. [52] demonstrated sensitive GC-based determination of MeHg in shrimp, tilapia, catfish, and water snake using a DB-5MS column, achieving an LOQ of 0.012 μg/kg.

#### 3.3.3. Capillary Electrophoresis (CE)

CE is another chromatographic technique, which has been employed in mercury speciation of seafood (Table 1), most commonly relying on L-cysteine to form charged Hg complexes suitable for electrophoretic separation [81]. A gradient mixture of boric acid combined with MeOH [79] or sodiumtetraborate [39,40] or more complex systems incorporating boric acid, EDTA, and CTAB solutions can be used as a running separation buffer [34].

Despite its advantages in terms of separation efficiency and low reagent consumption, CE-based mercury speciation faces intrinsic sensitivity limitations. UV detectors, which are most widely available for CE, are poorly suited for mercury analysis due to the weak UV absorbance of Hg species [40]. Organomercury compounds like MeHg and EtHg lack chromophores and are not detectable in their native form, consequently derivatization using chromogenic compounds is frequently necessary. Choosing suitable derivatizing reagents is thus crucial for effective CE separation and UV detection, and they must balance water solubility, complex stability, and detectability.

Cao et al. [45] compared ammonia pyrrolidine dithiocarbamate and thiosalicylic acid as derivatizing agents, finding ammonia pyrrolidine dithiocarbamate hindered CE separation while thiosalicylic acid enabled effective UV detection of organomercury species. Their CE–UV method achieved LODs of 76.4, 83.0, and 76.9 μg/L for MeHg, EtHg, and PhHg, respectively. Substantially improved sensitivity has been achieved by coupling CE with ICP-MS. Chen et al. [34] developed a CE–ICP-MS method exploiting selective binding of iHg and MeHg to specific DNA sequences, resulting in conjugates with different charge densities and enabling rapid separation within 11 min, with LODs of 0.12 and 0.10 μg/L for iHg and MeHg, respectively.

### 3.4. Detection Techniques

Detection strategies for mercury speciation in seafood are selected based on sensitivity, selectivity, cost, analysis time, and matrix compatibility. For LC-based separations, atomic spectrometric detectors—particularly ICP-MS and AFS—are most commonly employed due to their element-specific response. GC separations have been successfully coupled with a wide range of detectors, including electron capture detection [15], MS [23,36], ICP-MS [27,82], CVAFS [51,54] and DBDOES [25]. Meanwhile, ICP-MS, UV, and DAD are the most frequently used detectors in CE [9].

Among all detection techniques, ICP-MS and AFS are widely regarded as reference detectors for mercury speciation due to their high sensitivity and elemental specificity. ICP-MS, in particular, offers exceptionally low LODs and supports isotope-selective detection, enabling advanced quantification strategies such as isotope dilution [1]. However, both ICP-MS and AFS introduce challenges from a green analytical chemistry perspective. Derivatization requirements in AFS and extensive clean-up procedures needed to minimize interferences in ICP-MS can increase reagent consumption and analytical complexity.

A key limitation of hyphenated chromatographic techniques is the need to maintain compatibility between the separation system and the detector. For example, the composition of the eluent can significantly impact the efficiency of ICP-MS. Organic solvents, such as MeOH, have been shown to induce carbon deposition on the ion lenses, sampling and skimming cones or cause instability in plasma discharge [26]. Chen et al. [83] addressed these issues by increasing plasma forward power, cooling the spray chamber, and introducing oxygen-containing gas to counteract carbon buildup. Their approach significantly improved signal-to-noise ratios, and even after continuous operation with mobile phases containing up to 35% MeOH and 40% acetonitrile for 8 h, minimal carbon deposition was observed. While such mitigation strategies enhance robustness, they inevitably increase instrumental complexity and operational cost.

### 3.5. Biosensors

**Figure 3:**
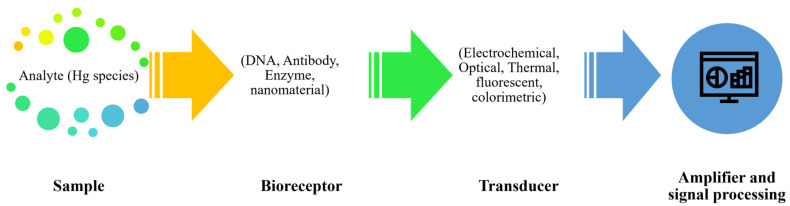
Working principle of Hg biosensors.

Despite the high analytical reliability of chromatographic methods, their complexity and limited suitability for rapid or on-site analysis have motivated the development of biosensor-based approaches for mercury species detection. Biosensors integrate a recognition element with a signal transduction system to enable selective detection of target analytes [84]. As shown in Figure 3, the functionality of biosensors hinges on two main components: the molecular recognition material and the signal recognition and analysis system. Different molecular recognition materials have been tested for Hg species, such as enzymes, antibodies, nucleic acids, or whole cells, which interact specifically with the target analyte [85]. This interaction generates a signal that is captured and processed by the signal recognition and analysis system, which converts the biological response into a measurable output. By optimizing these components, biosensors could achieve high sensitivity and specificity, enabling the detection of mercury species at low concentrations with greater speed and lower cost compared to traditional methods [84,86].

Although sensors for measuring iHg or THg have been developed, most MeHg sensors remain at a proof-of-concept stage, with limited validation in complex seafood matrixes and insufficient comparison to chromatographic reference methods [87]. The following section is an overview of some of the important mercury detection sensors, including colorimetric sensors, electrochemical sensors, and fluorescent sensors, that have been developed for the analysis of Hg species in seafood and summarizes the main results shown in Table 4.

#### 3.5.1. Electrochemical-Based Biosensors

Electrochemical biosensors detect mercury species through analyte-induced changes in electrical signals at the electrode interface, typically monitored as variations in current, potential, or impedance during oxidation–reduction processes [84,99]. The measured current reflects the rate of electron transfer associated with the redox activity of the target analyte. These platforms are widely regarded as highly sensitive, selective, rapid, and reliable, making them particularly attractive for trace-level mercury analysis [99].

In addition to biorecognition-based electrochemical sensors, unmodified solid gold electrodes (SGEs) have long been employed for Hg determination based on the strong affinity between Hg and gold, leading to the formation of a gold–Hg amalgam. In stripping-based techniques, Hg species are first preconcentrated onto the gold surface and subsequently quantified by anodic stripping voltammetry, providing well-defined stripping peaks and low detection limits in aqueous matrices and fish digests [100]. More recently, SGE-based systems coupled with selective SPE have demonstrated the feasibility of portable MeHg determination in fish samples, integrating matrix clean-up with on-site voltammetric detection [101]. Although SGEs do not incorporate biological recognition elements, they represent a robust and field-deployable electroanalytical platform that complements biosensor-based approaches for Hg speciation.

Recent electrochemical biosensors frequently employ nanomaterials or DNA-based recognition elements to improve sensitivity. For instance, Ran et al. [98] developed a thymine-mediated electrochemical aptasensor capable of simultaneous detection of inorganic mercury (iHg) and methylmercury (MeHg) in fish muscle. The sensor exploits the specific thymine–Hg interaction to induce conformational changes in DNA, leading to a decrease in electrochemical signals from ferrocene (Fc) and methylene blue (MB) tags. This dual-signal strategy enabled ultralow limits of detection of 0.06 pM for iHg and 0.13 pM for MeHg without requiring prior separation steps. Importantly, the results showed strong agreement with HPLC–ICP-MS, confirming the analytical reliability of the approach. Similarly, El-Raheem et al. [96] developed an electrochemical sensor based on poly(ester-urethane) urea (PUU) doped with gold nanoparticles (PUU/AuNPs) for iHg detection in fish. PUU offers strong binding affinity for iHg, while the gold nanoparticles enhance conductivity and signal response. The sensor exhibited a low detection limit of 0.235 µ/g L, good selectivity, and minimal sample preparation, highlighting its potential for practical seafood analysis.

Enzyme-inhibition-based biosensors are among the earliest and most established approaches for MeHg determination in fish and seafood. These systems exploit the strong affinity of mercury species for sulfhydryl groups, leading to inhibition of enzymatic activity that can be transduced electrochemically. Commonly used enzymes include urease, glucose oxidase, and horseradish peroxidase [85]. In this context, Amine et al. [102] developed an electrochemical method based on invertase inhibition to detect MeHg in fish tissue. MeHg forms a complex with invertase in an organic/aqueous system, reducing glucose production. The resulting decrease in hydrogen peroxide generation is monitored via oxidation at a platinum electrode, allowing selective discrimination of MeHg from other heavy metals.

Despite the remarkable sensitivity and innovation demonstrated by electrochemical biosensors, many studies remain focused on aqueous samples, with comparatively limited validation in complex seafood matrices. This highlights an important gap that future research must address to ensure robust, real-world applicability in food safety monitoring.

#### 3.5.2. Colorimetric Biosensors

Colorimetric biosensors enable visual or spectrophotometric detection based on analyte-induced color changes, offering sensitivity, affordability, and rapid detection without advanced instruments. In mercury speciation, their applicability is constrained by limited selectivity among Hg species and susceptibility to matrix interferences.

To improve these sensors, researchers are using techniques like plasmonics and photonics, incorporating metal nanoparticles (gold, copper, iron, platinum, palladium) for enhanced performance in biomedical and environmental analysis [84]. As mentioned in Table 4, Huang et al. [86] developed a DNAzyme-based colorimetric sensor that integrates task-specific ionic liquids (TSILs) for sensitive iHg detection in solid food samples. In this system, TSILs first extract iHg from microwave-assisted acid digestion solutions, followed by the addition of peroxidase-mimicking DNAzymes (PMDs). Residual PMDs catalyze the colorimetric oxidation of ABTS to its green radical cation (ABTS^+^), with the signal intensity proportional to the iHg concentration. This approach achieved a detection limit of 0.1 µg/L, representing a significant improvement over earlier DNAzyme-based colorimetric sensors.

Although analytically innovative, the requirement for digestion and extraction steps limits the suitability of such systems for rapid or truly on-site seafood analysis. In a more straightforward approach, Saranchina et al. [89] developed a colorimetric optode for the preconcentration and visual detection of iHg in fish products. The sensor is based on a color reaction between copper diethyldithiocarbamate and mercury ions embedded in a polymethylmethacrylate matrix, producing a visible color change from brown to colorless. The method showed good linearity in the range of 20–360 µg/L, with a detection limit of 8 µg/L and a response time of approximately 10 min.

While this approach is less sensitive than TSIL-assisted systems, its simplicity, low cost, and visual output without the need for instrumentation make it highly practical for rapid screening applications, especially in low-resource settings. Overall, colorimetric biosensors are best suited for preliminary screening, but their limited species discrimination and matrix robustness restrict their use in quantitative mercury speciation.

#### 3.5.3. Fluorescence-Based Biosensors

Fluorescence-based biosensors have attracted interest due to their high sensitivity and potential for selective mercury detection [91]. They measure the light emitted by a substance when its electrons move from an excited state back to the ground state. Several fluorescence-based sensors have demonstrated detection of organomercury species at the low ppb level under controlled conditions [84]. Recent research focuses on the development of fluorescent chemosensors using organic dyes, quantum-dot materials, and metallic nanomaterials for optical imaging and sensing [99,103].

However, fluorescence measurements in seafood matrices are often complicated by autofluorescence, quenching effects, and signal drift, which can hinder accurate quantification. Shen et al. [92] addressed some of these challenges by developing a fluorescence-polarization-based biosensor for total mercury (THg) determination in canned fish. The system employs thymine-rich single-stranded DNA immobilized on magnetic nanoparticles and exploits the thymine–Hg^2+^–thymine interaction to induce hybridization with a fluorophore-labeled aptamer. This process increases the effective molecular weight of the fluorophore, leading to enhanced fluorescence polarization. The sensor exhibited a linear response over 0.4–200 µg/L, with a detection limit of 0.09 µg/L, and could be reused for up to six cycles.

In a different strategy, Deng et al. [93] introduced a highly selective and sensitive fluorescence method for detecting MeHg using DNA-protected silver nanoparticles and a MeHg-specific probe. This technique exploits the amalgamation reaction between silver and mercury to discriminate MeHg from iHg, enabling detection down to the picomolar level and showing strong selectivity even in the presence of high iHg or other metal ions. While this method offers exceptional sensitivity and selectivity, its reliance on nanoparticle synthesis and stability may require careful optimization for routine analysis.

More recently, Erdemir et al. [91] developed a novel fluorescent probe combining triphenylamine (TPA), thiophene, and thiosemicarbazide (TSC). The probe exhibited an ultralow detection limit (<0.04 µg/L), a rapid response time (<30 s), and successful application to real seafood samples, including seabass and swordfish. While notable for its simplicity and speed, further validation across a broader range of seafood matrices and environmental conditions is still needed.

Despite their analytical advantages, fluorescence-based biosensors face challenges such as photobleaching, background autofluorescence, and long-term signal stability when applied to real seafood samples. Additionally, the use of complex synthesis routes or potentially hazardous reagents may limit their widespread adoption in routine food safety testing. Moreover, although biosensors demonstrate promising sensitivity, their application to real seafood matrices remains challenged by matrix interferences associated with complex protein and lipid compositions. Moreover, validation against CRM is still limited, and reproducibility data are often restricted to single-laboratory studies. These factors currently limit regulatory readiness compared to well-established chromatographic methods.

### 3.6. Comparative Analytical Performance of Chromatographic Versus Sensor-Based Approaches

Clear differences are observed between chromatographic techniques and sensor-based methods when comparing limits of detection (LODs) and analysis time. LC-based methods, particularly LC–ICP-MS, provide the lowest method LODs for mercury speciation in seafood, typically in the range of 0.01–1 µg kg^−1^, with chromatographic run times generally between 3 and 20 min, depending on column chemistry and mobile phase composition. GC-based approaches achieve comparable sensitivity, but the overall time required for analysis is substantially longer due to mandatory extraction and derivatization steps. Total analysis times commonly range from 50 min to several hours, despite relatively short GC separation times. CE-based methods often exhibit higher detection limits when UV or DAD detection is used, whereas CE–ICP-MS achieves substantially lower LODs, which restricts their applicability to samples with elevated mercury concentrations or to screening purposes.

Sensor-based methods exhibit a distinct performance profile. Reported instrumental LODs in solution typically fall between 0.04 and 8 µg/L, with response times from seconds to minutes. However, when applied to seafood matrices, additional extraction or digestion steps are often required, reducing the effective time advantage and limiting direct comparison with chromatographic workflows. The suitability of each analytical approach may also depend on seafood type, particularly for matrices with high lipid content (e.g., fish liver, fish oil) or complex protein structures (e.g., shellfish), where extraction efficiency becomes the dominant factor influencing performance.

## 4. Conclusions

This systematic review critically evaluated chromatographic and sensor-based approaches for mercury speciation in seafood. Hyphenated chromatographic techniques remain the backbone of mercury speciation analysis, but analytical reliability is strongly governed by sample preparation, which continues to be the main source of uncertainty. Despite major advances, no single chromatographic approach can be regarded as universally optimal, as performance depends on matrix type, extraction strategy, and analytical purpose. Among the available strategies, LC–ICP-MS generally achieves the lowest detection limits and highest analytical robustness, although it requires advanced instrumentation and higher operational costs compared to sensor-based alternatives. Sensor-based methods address the need for faster and simpler measurements, yet their application in seafood is still limited by matrix effects and the frequent need for prior extraction. For this reason, sensors are best viewed as supporting tools for screening and rapid decisions, while chromatographic methods remain essential for definitive speciation and reliable quantification. Together, both approaches can play complementary roles in seafood safety monitoring when applied within clearly defined analytical contexts. Thus, the choice of method ultimately reflects a trade-off between analytical robustness and operational simplicity, depending on whether confirmatory accuracy or rapid screening is the primary objective.

## 5. Future Perspectives

Future research on mercury speciation in seafood is expected to follow two parallel directions. For chromatographic methods, the main needs lie in improving standardization, particularly for extraction procedures, evaluation of species stability, and validation across different seafood matrices. Addressing these aspects would strengthen method comparability and support wider routine application. In contrast, most biosensor studies remain focused on inorganic mercury in water samples, and the detection of organomercury species—especially methylmercury—in seafood is still relatively limited. Progress in this area will require sensors that demonstrate reliable performance in complex matrices, alongside clearer validation practices and comparison with established analytical methods.

## Figures and Tables

**Table 1:** Extraction procedures and chromatographic conditions adopted for mercury speciation in seafood.

n.	Type of Seafood	Analytes	Sample Prepa Ration	Sample Prep Time	Analytical Method	Conc in Sample	Chromatographic Column	Mobile Phase	LOD (µg/kg, Method)/(µg/L, Instrumental)	Recovery	Linearity Range	Used CRM	Analysis Time	References
1	CRM	iHg MeHg	Repeated extraction with HCL and 2-ME, centrifugation, buffering with NH_4_Ac then filtration	150 min	HPLC-ICP-MS	iHg: 30–290 µg/kg MeHg: 1–4280 µg/kg	Column:Purospher^®^ STAR RP-8e, 75 × 4 mm, 3 μm Guard column: Purospher^®^ STAR RP-8e, 4 × 4 mm, 5 μm	0.02 M NH_4_Ac, 0.2% 2-ME, 1% MeOH	–	MeHg: 98–102%	0.01–0.05 μg/L	DORM-2 CRM-422 NIST SRM 2976 SRM-1570a CRM-185	12 min.	[14]
2	Fish (chub muscle)	MeHg	Acidic digestion and extraction with toluene	–	GC-ECD	MeHg: 65–231 µg/kg	Capillary column DB 608 (30 m × 0.53 mm × 0.83 µm)	Helium	21 µg/kg	MeHg: 89 ± 2.5%	–	BCR-463 BCR-464	–	[15]
3	24 seafood samples	iHg MeHg	MAE with HCl	<1 h	LC-UV-CV-AFS	MeHg: 3–2230 µg/kg iHg: 6–85 µg/kg	Analytical RP-C18 column (ODS Hypersyl 250 mm A 4.6 mm id, 5 mm, Thermo Hypersil-Keystone)	20% APDC and NH_4_Ac solution and 80% MeOH	iHg: 0.4 µg/kg MeHg: 0.3 µg/kg	MeHg: 80–102%	–	DOLT-4 TORT-2 BCR-463	8 min.	[4]
4	12 species of commercial fish in Cuba	iHg MeHg	UAE: 0.1% 2-ME, 0.05% L-cysteine and 0.1% HCl	30 min	ID-LC-ICP-MS	MeHg: 40–1920 µg/kg iHg: <62 mg/kg	Tracer Spherisorb C8 5 μm (100 mm × 4.6 mm)	0.05% 2-ME, 0.075% L-cysteine and 0.06 M NH_4_Ac buffer	MeHg: 7.7 µg/kg iHg: 5.2 µg/kg	MeHg: 93–99%	–	DOLT-2 DORM-2	<10 min	[16]
5	Cod, tuna, mackerel, and bonito	MeHg	Extraction with acetone, toluene; treatment of residue with KBr, copper sulfate, sulfuric acid. Treatment of toluene layers with L-Cys, HCl; derivatization of 4 mL aliquot with phosphate buffer, NaBPh_4_	50 min	GC-MS	MeHg: 150 µg/kg	InertCap 5MS/NP (0.25 mm i.d. × 30 m long, 0.25 μm) column	Helium	–	80–110%	–	CRM-7402a BCR-463 BCR-464 CRM-7403a	9 min	[17]
6	6 fish species (bearded brotula, bluewing searobin, flatfish, pirarucu, salmon, and yellowfin tuna)	iHg MeHg	Extraction with 6 mL of L-Cys, centrifugation, and filtration	1–18 h	LC-CVG-ICP-MS	MeHg: <500 µg/kg	Si-C18 RP-column (Discovery, 250 × 4 mm) and guard column Si-C18 (Allchrom, 4 × 3 mm), both with particle size of 5 μm	0.1% L-cysteine solution at pH 4	iHg: 1.7 µg/kg MeHg: 2.3 µg/kg	97–102%	0.10–5 μg/L	DORM-2 DOLT-3	6 min	[18]
7	Long-finned pilot whale samples (liver, kidney and muscle)	MeHg	MAE in 2 mL TMAH (60 °C). Ultracentrifugation, dilution of 1 mL clear digest in water, acidification with HCl, topped up to 50 mL	40 min	HPLC-CV-AFS and species-specific isotope dilution GC-ICP-MS	MeHg: >1000 µg/kg by GC	Eclipse XDB C8 (4.6 × 150 mm, 5 μm) for HPLC; 100% PDMS Silcosteel^®^ (30 m × 0.59 mm × 1.0 μm) for GC	75% MeOH, 1.5 mM APDC (HPLC), helium (GC)	0.0015 μg/L (HPLC)	88–104%	0–0.5 μg/L (HPLC)	DOLT-2 DOLT-4 TORT-2 DORM-2	15 min	[19]
8	Fish (Black Sea)	iHg MeHg	Homogenized with 0.15% 2-ME, 0.01 M HCl, microwaved (60 °C), centrifugation, filtration	2 min	HPLC-ICP–MS	<500 µg/kg for muscle meat of fish and fishery products	Zorbax C18 (100 × 4.6 mm, 3.5 μm)	55.0% MeOH, 0.1% 2-ME, 60 mM NH_4_Ac, pH 4.0	iHg: 0.2 µg/kg MeHg: 0.1 µg/kg	97.6–98.5%	0.0–20.0 μg/L	TORT-2 DORM-2	4 min	[20]
9	Silver carp muscles (pelagic fish)	iHg MeHg EtHg PhHg	Sonication with 5 M HCl, centrifugation. Second extraction, combination of supernatants, adjusted to pH 7 with NaOH, diluted with water	80 min	HPLC-ICP-MS	MeHg, iHg: from 9–88.2 µg/kg, EtHg, PhHg: not detected	C18 column (Lichrospher- ODS, 200 × 4.6 mm, 5 μm)	MeOH-buffer solution (2.5 mM L-Cys, 12.5 mM (NH_4_)_2_HPO_4_, 0.05% triethylamine, pH 7.0)	iHg:0.0041 μg/L MeHg: 0.0029 μg/L EtHg: 0.0047 μg/L PhHg: 0.0056 μg/L	MeHg: 92.67%	iHg, EtHg, PhHg: 0.02–5 μg/L MeHg: 0.01–5 μg/L	DORM-2	13.3 min	[21]
10	Grouper, pufferfish	iHg MeHg	MWCNT-assisted MSPD extraction: Blended 4 mg MWCNTs, 10 mg sample. Transferred mixture with 0.20 g C18 co-sorbent to polypropylene column	35 min	MSPD-HPLC-ICP-MS	iHg: 0.047 µg/kg (grouper), 0.016 µg/kg (pufferfish). MeHg: 0.277 µg/kg (grouper), 0.193 µg/kg (pufferfish)	Agilent zorbax SB-C18 (4.6 mm i.d. × 250 mm, 5 μm)	8% MeOH, 0.12% L-Cys, 10 mM NH_4_Ac, pH 7.5	iHg: 9.9 μg/L MeHg: 8.4 μg/L	iHg: 94% MeHg: 106%	–	DORM-2 DORM-3		[22]
11	Canned tuna in water and oil, 6 types of commercial seafood, 2 frozen fish (*Merluccius merluccius* and *Salmo salar*)	MeHg EtHg	Sonication with methanolic KOH. Adjusted pH with AcOH-NaOAc buffer. Added 0.8 mL NaBPh_4_ (2.0% m/v) and 2 mL hexane	90 min	GC-MS	MeHg: <LOD in most samples, while those quantified were <1000 µg/kg for predator fish and <500 µg/kg for the others. EtHg: not detected	Thermo non-polar TG-5MS (30 m × 0.25 mm I.D., 0.25- μm) capillary column (5% diphenyl, 95% dimethylpolysiloxane)	Helium	MeHg: 1.61 µg/kg EtHg: 1.95 µg/kg	70–120%	20–900 μg/L	NIST SRM 2976 CRM-7402a	9 min	[23]
12	7 seafood products (muscle tissue, squid muscle, crab claw meat, whale meat, cod muscle, halibut muscle and dogfish liver)	MeHg	Digestion: TMAH overnight for complete dissolution. Buffer addition: 1 mL pH 5 AcOH-NaOAc buffer, 600 μL HNO_3_. Derivatization: 1 mL hexane, 500 μL NaBEt_4_, centrifugation	–	GC–ICP-IDMS	MeHg: 35–3580 µg/kg (as Greenland halibut muscle had the highest concentration)	30 m × 0.32 mm id column with HP-5–5% phenyl methyl siloxane; 0.25 μm	Helium	–	99.9%	50–500 μg/kg	NIST-SRM1566 BCR-464 DOLT-4	–	[24]
13	CRM	iHg MeHg EtHg	Derivatization: with NaBPh_4_, preconcentrated via headspace SPME with porous carbons. CRMs treated with 25% KOH-MeOH, heated (65 °C), diluted to 25 mL with MeOH	4 h	GC-DBD-OES	nd	HP-5 capillary column (30 m length × 0.32 mm i.d. × 0.25 μm)	Argon	iHg: 0.5 µg/kg MeHg: 0.75 µg/kg EtHg: 1 µg/kg	90–105%	iHg: 0.5–30 μg/L MeHg: 0.5–50 μg/L EtHg: 1–100 μg/L	DORM-4 TORT-3	10 min	[25]
14	Bream and crucian fish	iHg MeHg PhHg	MSPE using Fe_3_O_4_@SiO_2_@GMA-MPTS as sorbents	20 min	MSPE-HPLC-ICP-MS	MeHg: 35.6 µg/kg and 19.1 µg/kg in bream and crucian	C18 column (Hypersil ODS2, 250 × 4.6 mm, 5 μm)	50:50 MeOH-buffer solution (0.1% 2-ME, 50 mM NH_4_Ac, adjusted to pH 5 with 0.1 M HAc)	iHg: 0.0007 μg/L MeHg: 0.0007 μg/L PhHg: 0.0005 μg/L	iHg: 94–106% MeHg: 95–105% EtHg: 86–94%	0.005–5 μg/L	DORM-2	8 min	[26]
15	Meat samples of 84 minke whales	MeHg	Sample digested with TMAH, pH adjusted using NaAc/HNO_3_, derivatized with NaBEt_4_. EtHg extracted into hexane	–	GC–ICP–MS	MeHg: 140– 230 µg/kg	ICSep ION-120 ion exchange column	–	MeHg: 30 µg/kg	96–102%	–	TORT-2	–	[27]
16	Tuna	iHg MeHg EtHg	Mixed, shaken, CH_2_Cl_2_ added, titrated with HCl. Phases separated, CH_2_Cl_2_ transferred to a glass tube. Sodium thiosulfate added, shaken, and centrifuged	–	HPLC-DBD-plasma-CVG-AFS	–	Zorbax SB-C18 (4.6 mm × 75 mm, 3.5 μm)	0.01% 2-ME, 10% MeOH and 0.06 M NH_4_Ac at pH 6.8	iHg: 1.6 μg/L MeHg: 0.42 μg/L PhHg: 0.75 μg/L	iHg: 98.8% MeHg: 92.8% EtHg: 91.9%	5–200 μg/L	GBW10029	<14 min	[28]
17	Three fish oil and fish liver samples	iHg MeHg EtHg	MAE with 2-ME, MeOH, lipase, Triton X-100, HNO_3_ at 60 °C, cooled, and centrifuged	5 min	HPLC ICP-MS		RP-C8 column PerkinElmer C8 (3.0 mm × 33 mm; 3- μm)	0.6% 2-ME, 3% MeOH	0.013–0.026 μg/L	93–107%	0.2–10 μg/L	DOLT-3	6 min	[29]
18	Fresh fish, canned fish	iHg MeHg EtHg	Sonication with HCl, centrifugation, pH adjusted to 2.0 with a NaOH and citric acid/citrate	1 night and 4 h	HPLC-CV-AFS	MeHg: 0.17– 0.26 mg/g iHg: 0.14 mg/g EtHg: 0.13– 0.19 mg/g in canned seafood MeHg: 0.15–0.25 mg/g iHg: 0.13 in fresh seafood	Thermo Scientific–Hypersil GOLD aQ–C18 column (150 mm × 4.6 mm i.d.)	A: 0.4% 1-octyl-3-methylimidazolium chloride [C8mim] Cl,100 mM NaCl, and 20 mM buffer citric acid/citrate at pH 2.0 B: MeOH	iHg: 0.1 μg/L MeHg: 0.05 μg/L EtHg: 0.06 μg/L	94.6–99.2%	iHg: 0.4–1000 μg/L MeHg EtHg: 0.2–1000 μg/L	–	12 min	[30]
19	CRM	iHg MeHg EtHg	Heat-assisted alkaline extraction with TMAH at 80 °C	2 h	HPLC-ICP-MS	MeHg: 58–5100 μg/kg	(i) Octadecylsilyl column (VP-ODS5 μm, i.d. 4.6 × 250 mm), (ii) Adamantyl column (ADME, 3 μm, i.d. 4.6 × 150 mm)	0.5 g/L L-Cys/1% MeOH (pH 2.3 with HCl)	MeHg: 0.08 and 0.13 µg/kg for ADME and VP-ODS5 columns	MeHg: 101% iHg: 103%	Up to 50 µg/kg	CRM-7402a CRM-7403a DORM-2 NIES CRM No. 13	6 and 4 min for ADME and VP-ODS5 columns	[31]
20	CRM	iHg MeHg	UAE: 5 M HCl	15 min	SPE-HPLC-ICP-MS	iHg: 18–161 μg/kg MeHg: 141– 3130 μg/kg	Phenomenex SphereClone ODS C18 (5 mm, 250 mm 4.6 mm i.d.)	0.5% formic acid; 0.2% 2-ME; 20% MeOH	iHg: 0.015 μg/L MeHg: 0.017 μg/L	>93%	0.01–10 μg/L	BCR 643 TORT-3	10 min	[32]
21	Freshwater fish	iHg MeHg EtHg PhHg	UAE: 40 °C, 5 M HCl, 2 mm L-cysteine	15 min	IP-RPC-ICP-MS	MeHg: <0.6 μg/g iHg: 3.7–2915 μg/g EtHg: <1.1 μg/g PhHg: <1.7 μg/g	Zorbax C18 25 mm × 4.6 mm × 5 μm	2.0 mM SDBS, 2.0 mM Cys, 1.0 mM Phe (pH 3.0) and 4.0 mM TBAH, 2.0 mM MPS, 2.0 mM Phe (pH 6.0)	iHg: 0.02 μg/L MeHg: 0.01 μg/L EtHg: 0.03 μg/L PhHg: 0.04 μg/L	iHg: 94–104% MeHg: 93–106% EtHg: 91–104% PhHg: 95–101%	0.1–100 µg/kg	GBW10029 BCR-463	3 min	[33]
22	Dried fish muscle	iHg MeHg	Sample was 50 μm DNA (HT7 sequence), agitated, then diluted to 0.5 mL with buffer	15 min	CE–ICP-MS	iHg: 0.018–0.161 μg/g MeHg: 0.473–0.9723 μg/g		0.72 mM Tris buffer, 0.72 mM boric acid, 16 mM EDTA, and 0.3 mM CTAB (pH = 8.0)	iHg: 0.12 μg/L MeHg: 0.10 μg/L	95–101%	5.0–200 μg/L		11 min	[34]
23	Fish tissue	iHg MeHg EtHg PhHg	UAE with L-cysteine in HCl, 40 °C, centrifuged, neutralized	15 min	HPLC-ICP-MS	MeHg: 0.81–0.04 mg/kg iHg: 0.018–0.004 mg/kg	GSiO_2_ column (graphene oxide on silica)	NH_4_Ac, 2-thiosalicylic acid, Phe, and positively charged BHDMA	iHg: 0.016 μg/L MeHg: 0.027 μg/L EtHg: 0.032 μg/L PhHg: 0.068 μg/L	92–96%.	0.5–500 μg/L	GBW10029	12 min	[35]
24	40 canned tuna samples from Tehran, Iran (white and light style)	THg MeHg	–		GC-MS	MeHg: 207– 352 μg/kg iHg: 111–172 μg/kg in light tuna MeHg: 88–135 μg/kg iHg: 136–303 μg/kg in white tuna	CP-Sil5CB (100% polydimethylsiloxan) fused-silica capillary column (25 m × 0.32 mm i.d. and 1.2 μm)	Helium gas	iHg: 3 µg/kg MeHg: 5 µg/kg	–	–			[36]
25	Fish tissues	iHg MeHg	MAE with HNO_3_ and HCl		HPLC and CVG MC-ICPMS	MeHg: 229.05 μg/kg in white tuna, 113.37 μg/kg in light-style tuna	C18 column (PROTECOL C18G123 100 × 10 mm)	0.05% m/m L-Cys, 0.006% v/m AcOH adjusted to pH 4 with ammonia	–	–	–	NIST SRM 1947 BCR-463	15 min	[37]
26	Sea cucumber (*Apostichopus japonicus*)	iHg MeHg EtHg	UAE with HCl, L-cystein and 2-ME	30 min	HPLC-ICP-MS	iHg: 0.04–1.9 mg/kg MeHg: 0.04–2.5 mg/kg	Agilent Zorbax SB-C18 analytical and guard columns	8% MeOH and 92% H_2_O	iHg: 0.12 μg/L MeHg: 0.08 μg/L EtHg: 0.20 μg/L	91.3–104.1%		GBW10024	10 min	[38]
27	Fish muscle and kelp samples		Sonication in 5 HCL		CE-DAD	0.038–0.054 mg/mL	Bare fused-silica capillary	Sodium tetraborate	11–16 μg/L	84.63–111.39% for fish and 75.68–114.76% for kelp	50–2000 μg/L			[39]
28	Fish tissues (Chinese rare minnow)	MeHg EtHg PhHg	Alkaline extraction, dissolved in Na_2_S_2_O_3_		CE-UV			30 mM borate buffer at pH 9.2	MeHg: 76.4 μg/L EtHg: 83.0 μg/L PhHg: 76.9 μg/L		400–2000 μg/L	DORM-2	25 min	[40]
29	Two fish samples	iHg MeHg EtHg PhHg	Alkaline extraction with TMAH and HCl at 75 °C	30 min	HPLC-UVPVG and AAS detector	iHg: 0.05–1.16 mg/kg MeHg: 0.4–3.63 mg/kg	Gemini column (C18, 110 A, 3 lm, 150 mm length, 2.4 mm i.d.)	Organic solvent (MeOH, acetonitrile, and ethanol), buffer system (AcOH and NH_4_ or NaOAc), and photochemical reagent (2-ME)	iHg: 0.47 μg/L MeHg: 0.84 μg/L EtHg: 0.80 μg/L PhHg: 2.0 μg/L	–	Up to 100 μg/L	DOLT-4 BCR-464		[41]
30	Environmental water and fish samples	iHg MeHg	MSPE	–	MSPE-HPLC-ICP-MS	iHg: 0.12–0.18 mg/kg MeHg: 0.4–3.63 mg/kg	C18 column (Hypersil ODS2, 250 × 4.6 mm, 5 m particle size)	1.0 g/L L-Cys, 60 mM NH_4_Ac and 2% (vv) MeOH	iHg: 0.00048 μg/L MeHg: 0.00017 μg/L	iHg: 96–103% MeHg: 95–97%	iHg: 0.000016–0.005 µg/L MeHg: 0.0006–0.5 µg/L	DORM-4	–	[42]
31	Water, soil, rice and fish	iHg MeHg PhHg	MSPE using Fe_3_O_4_@SiO2@GMA-S-SH as sorbents	7 min	MSPE-HPLC-ICP-MS	iHg: 0.62 mg/kg MeHg: 4.11 mg/kg	Shim-pack CLC-ODS C18 column (15.0 mm × 6.0 mm × 5.0 mm)	8.0 mM L-Cys, 12.5 mM (NH_4_)_2_HPO_4_, 0.05% triethylamine, pH 7.0, MeOH (8:92)	iHg: 0.0004 μg/L MeHg: 0.00049 μg/L PhHg: 0.0014 μg/L	84.3–116%	0.005–5 µg/L	DORM-2	17 min	[43]
32	Clam, oyster and fish homogenate	iHg MeHg	Solid–liquid extraction with magnetic stirring: samples spiked with isotopically enriched MeHg then extracted with 15% HCl at 50 °C	40 min	HPLC and species-specific isotope dilution ICP-MS	–	Symmetry Shield RP C18, 150 × 3.9 mm, 5 μm and Guard column 3.9 mm × 5 mm, 5 μm	2-ME, 0.4% L-Cys, 0.06 M NH_4_Ac and 2% MeOH	iHg: 0.15 µg/kg MeHg: 0.42 µg/kg	–	–	IAEA-461 IAEA-470 IAEA-476	5 min	[44]
33	CRM	iHg MeHg	Anion exchange chromatography using AG 1-X4 that further isolates from the sample matrix, after a distillation treatment	–	MC-ICP-MS		AG 1-X4 anion exchange column	0.05% (*w*/*v*−1) L-cysteine and 1% citric acid trisodium salt dihydrate	MeHg: 5 μg/L	97 ± 4%	0.5–1 μg/L	DOLT-2 TORT-2		[45]
34	CRM	iAs iHg MeHg	UAE with TMAH at 80 °C	60 min	HPLC-ICP-MS	MeHg: 0.586 mg/kg	RP-C18 ODS column	5 mM sodium 1-butanesulfonate, 2 mM NH_4_H_2_PO_4_, 4 mM TMAH, 5 mM L-Cys, and 0.1% MeOH (pH 2.3)	MeHg: 0.09 µg/kg	94–102%	–	NMIJ CRM 740	12 min	[46]
35	Fish from the eastern Irish Sea	iHg MeHg Cu Pb Zn	MAE with HCl at 800 W and ion exchange chromatography	30 min	ID ICP-MS	MeHg: 0.53 mg/kg	Anion exchange column	–	–		–	IAEA-476		[47]
36	Canned tuna fish tissues	iHg MeHg	MAE with L-cysteine HCl·H_2_O at 60 °C, dilution with MeOH, centrifugation and filtration	15 min	HPLC-ICP-MS	MeHg: 0.500–0.800 g	RP-C18, Agilent peptide mapping (2.1 × 150 mm, 2.7 μm) and precolumn C18 poroshell (2.1 × 50 mm; 2.7 μm)	5% MeOH and 95% L-cysteine. HCl. H_2_O		80–120%	1–10 μg/L	DOLT-5 BCR-464 TORT-3	<3 min	[48]
37	Tuna fish and seaweeds	iHg MeHg	UAE with HCl, L-cysteine, 2-ME followed by IIP-SPE	60 min	HPLC-ICP-MS	iHg: 0.001 mg/kg MeHg: 0.05 mg/kg	Kinetex C18 100 Å (100 mm × 2.10 mm, 5 μm) connected with a C18 guard column	0.4% 2-ME, 10% MeOH, pH 2.0	iHg: 0.02 μg/kg MeHg: 0.007 μg/kg	iHg: 86–108% MeHg: 89–112%	iHg: 0–50 μg/L MeHg: 0–20 μg/L	BCR-463	8 min	[49]
38	Freshwater tilapia, seawater tilapia, swordfish, freshwater bass and flatfish	iHg MeHg As	MAE with HCl and protease XIV kept in a pool of water which was heated to 70 °C	70 min	HPLC-DRC-ICP-MS	iHg: nd–134 μg/kg MeHg: 11.7–133 μg/kg	Zorbax SB-Aq C18 column (5 μm, 4.6 mm i.d. × 250 mm)	A: 5 mM 1-octanesulfonate, 5 mM acetate buffer and 1% isopropyl alcohol (IPA) at pH 4.0 B: 2 mM L-cysteine in 1% IPA (pH 4)	MeHg: 0.013 μg/L iHg: 0.015 μg/L	97–103%	0.2–10 μg/L	DORM-3	4.5 min	[50]
39	*Carassius auratus*, *Aristichthys nobilis* and silver carp fish	MeHg	Digestion with KOH in MeOH at 75 °C, diluted by aqueous ethylation	3 h	GC-CVAFS	MeHg: 12.9–27.5 μg/kg	–	–	0.02 µg/kg	90–110%	–	TORT-2		[51]
40	Crucian and grass carp fish	iHg MeHg EtHg PhHg	UAE with HCl, centrifugation, filtration. Then MSPE (Fe_3_O_4_@BD-TpMA-S-SH MOP) was performed	60 min	HPLC-ICP-MS	MeHg: 3.89–7.95 μg/kg iHg: 51.6–71.9 μg/kg EtHg: nd PhHg: nd	C18 column (Hypersil ODS2, 250 × 4.6 mm, 5 μm)	8.0 mM L-cysteine, 12.5 mM (NH_4_)_2_HPO_4_, 0.05% triethylamine, pH 7.0, MeOH (8:92)	iHg: 0.00043 μg/L MeHg: 0.00055 μg/L EtHg: 0.00069 μg/L PhHg: 0.0011 μg/L	82.6–110%	iHg, MeHg, EtHg: 0.002–5 µg/L PhHg: 0.005–5 µg/L	DORM-2	1000 s (=16.67 min)	[52]
41	Sea perch and water	iHg MeHg	Sonication with HCl, centrifugation, filtration. Then MSPE: (Fe_3_O_4_@UiO66-SH)9, elute with HNO_3_ and thiourea	17 min	HPLC-ICP-MS	iHg: 8.41 MeHg: <LOD	A Pgrandsil-FE-C18 column (150 × 4.6 mm id, 5 μm)	0.1% L-cysteine, 0.5% MeOH and 10 mM NH_4_Ac mixture solutions, at pH 7	0.00007–0.00013 μg/L	91.4–95.1%	0.02–1 µg/L	GBW10029	6 min	[53]
42	Shrimp, tilapia, catfish, and water snake	MeHg	Digested with KOH/MeOH at 80 °C, centrifugation, filtration, 300 μL buffer added, and 40 μL derivatizing reagent (NaBEt_4_)	5 h–10 min	GC-CVAFS	MeHg: 2.51–254 μg/kg dw	DB-5MS capillary column (15 m × 0.25 mm × 0.10 μm)	Ar and N2	–	–	–	–	–	[54]
43	Three zooplankton samples, four scalloped hammerhead shark muscle samples and two milk shark muscle samples	MeHg	UAE with HCl, centrifugation	15 min	FC-ICP-MS	MeHg: 0.12–5 mg/kg	Short, homemade anion exchange column		1.6 µg/kg		0.024–10 µg/kg	BCR-463 BCR-464 BCR414 and DOLT-5	100 s	[55]
44	15 elasmobranch species	MeHg	Sonication with EtHg chloride in MeOH and HCl, NaCl added, centrifugation, toluene extraction, L-cysteine back-extraction, derivatization with CuSO4 and NaBPh_4_		Trace Ultra GC connected with Polaris Q MS	MeHg: 0.41–0.63 µg/g	A SPB-608 capillary column (30 m × 0.53 mm id., 0.5 µm)	Helium (99.99%)	0.03 µg/kg	96–101%		TORT-3		[56]
45	Seaweed, fish and shellfish	iHg MeHg EtHg	MAE with HNO_3_–L-cysteine at 120 °C, cooled, centrifuged	6.5 h	IC-HPLC-ICP-MS	iHg: 0.07–0.13 µg/g MeHg: 0.09–0.52 µg/g EtHg: <0.10 in fish iHg: 0.05–0.08 µg/g MeHg: 0.05–0.15 µg/g EtHg: <LOD in shellfish	Two consecutive strong cation guard columns (Zorbax 300SCX, 4.6 × 12.5 mm)	1.0 mM Cys in 5 mM HNO_3_	iHg, MeHg, EtHg: 0.02–0.05 μg/L	92–105%	0.5–10 μg/L	Fish (P40219)	20 min	[57]
46	Shark meat	MeHg	Sonication with L-cysteine in HNO_3_	30 min	HPLC-ICP-MS	MeHg: 0.22–1.22 mg/kg	C18 Hypersil Gold column	0.5% L-cysteine in 2% HNO_3_ and 100% MeOH	MeHg: 8.6 × 10^−7^ μg/L	96.90%	1–100 μg/L	DORM4	300 s	[58]
47	Rainbow trout, tuna, swordfish, and dogfish	iHg MeHg Se	MAE with protease XIV in buffer, pH adjusted with HNO_3_ and 2-ME, then diluted with water	60 min	HPLC-ICP-MS	MeHg: 0.026–1.77 mg/kg	Dionex IonPac™ CS5A A cation exchange column p4 × 250 mm, 9 μm	Mixture of MeOH 5%, 45 mM HNO_3_, 0.015%2-ME, and 1.5 mM sodium 3-mercapto-1-propanesulfonate	MeHg: 0.011 μg/L	–	–	ERM-CE101 ERM-BB422 CRM-7402a DOLT-5	15 min	[59]
48	Asian clams (soft tissue), megaloptera larvae (whole body), mayfly larvae (whole body), crayfish (muscle tissue), shiner (skinless fillet)	MeHg	Digested with HNO_3_, 60 °C, centrifuged, diluted	12 h	GC-CVAFS		Column containing anion exchange resin		MeHg: 0.00007 μg/L	93.4 ± 2.9%	0.0005–0.1 µg/L	DORM-3 TORT-2 DOLT-2 DOLT-5		[60]
49	Rice, fish, soil	iHg MeHg	MAE with HCl (2, 4, 5, 7.5 M) and KOH (10, 20, 25, 30%), various temp (50 to 125◦C)	50–60 min	HPLC-ICP-MS	MeHg: 0.68–2.26 µg/kg in fish	Zorbax Extended C18 column	0.06 M NH_4_Ac, 0.1% 2 -ME and 5% MeOH at pH 6.8	MeHg: 5 μg/kg	90–110%	0–20 μg/L	–	20 min	[61]
50	Fishery products	iHg MeHg	MAE with 2-ME and MeOH, diluted with water, filtered		HPLC-ICP-MS	iHg: 1.6–43.4 MeHg: 3.1–190.8 µg/kg	Advance Bio Peptide Map 2.7 μm, 2.1 × 150 mm	2-ME at 0.25%, MeOH at 1%	iHg: 0.005 μg/kg MeHg: 0.001 μg/kg	iHg: 80–120% MeHg: 90–120%	iHg: 0–1 μg/L MeHg: 0–5 μg/L	TORT-3 SRM 1566-b SQID-1 CRM-7402a	<8 min	[62]
51	Fish and shellfish (sea bream, shrimp, crab, scallop, salmon, sardine)	MeHg	QuEChERS-based extraction + HLB SPE + phenyl derivatization		GC–MS					86.1–98.3%		NMIJ CRM 7402-a, 7403-a, IAEA-436A		[63]
52	Fish, shrimp	MeHg, iHg, Cd, Sn, Pb species	Acid extraction with HCl followed by filtration	~30 min	HPLC–ICP-MS		Amphion II column	Aqueous solution containing sodium dodecylbenzenesulfonate and cysteine	0.08–0.10 µg kg^−1^ (MeHg)	86–105%	0.1–50 µg/L		~10 min	[64]

Abbreviations: 2,6-ditert-butyl-4-dimethylaminomethylphenol (BHDMA); cetyltrimethylammonium bromide (CTAB); cold vapor generation multi-collector ICP (CVG MC-ICPMS); Dynamic reaction cell (DRC); GC-electron capture detector (GC-ECD); Frontal chromatography (FC); HPLC and post-column ultraviolet-photochemical vapor generation (HPLC-UV-PVG); HPLC and dynamic reaction cell ICP-MS (HPLC-DRC-ICP-MS); ion-pairing (IP); liquid chromatography UV irradiation and cold vapor atomic fluorescence spectroscopy (LC-UV-CV-AFS); multi-wall carbon nanotubes (MWCNTs); assisted matrix solid phase dispersion (MSPD); phenylalanine (Phe); sodium 3-mercapto-1-propysulfonate (MPS); sodium dodecylbenzene sulfonate (SDBS); sodium acetate (NaOAc); tetrabutylammonium hydroxide (TBAH). CRM: BCR-463 (tuna fish); BCR-464 (tuna fish); BCR414 (plankton); CRM 422 (cod muscle); CRM 185 (bovine liver); DORM-2 (dogfish muscle); DORM-3 (fish protein); DORM-4 (fish protein); DOLT-2 (dogfish liver); DOLT-4 (dogfish liver); DOLT-5 (dogfish liver); ERM-CE101 (trout muscle); ERM-BB422 (fish muscle); GBW10029 (tuna fish); GBW10024 (scallop); IAEA-461 (clam); IAEA-470 (oyster); IAEA-476 (fish homogenate); NIST SRM1566b (oyster tissue); CRM-7402a (cod fish tissue); CRM-7403a (swordfish tissue); NIES CRM No. 13 (human hair); NIST SRM 2976 (mussel tissue); P40219 (fish); SRM 1570a (spinach leaves); TORT-2 (lobster hepatopancreas). nd: not detected.

**Table 2:** Advantages and disadvantages of different sample preparation techniques.

**Method**	**Advantages**	**Disadvantages**
MAE	- Rapid and reproducible extraction - High efficiency	- Laborious, expensive setup - Risk of sample degradation due to high temperatures
UAE	- Simple and cost effective	- Strong dependence on ultrasound intensity and sonication parameters, affecting extraction efficiency and species stability - Risk of sample degradation
SPE	- Simple, widely available - High selectivity - Good sample clean-up and preconcentration	- Limited sorbent capacity - Time-consuming - Large solvent volumes - High cost of advanced sorbents (CNTs, IIPs, MNPs)
SPME	- Reduced solvent - High sensitivity and selectivity - Simple and rapid	- Limited to volatile and semi-volatile compounds - Fiber fragility and limited adsorption capacity

**Table 3:** Principles, advantages, and disadvantages of chromatographic techniques used for mercury speciation analysis.

	Principle	Advantages	Disadvantages
LC	Analytes separate depending on polarity, solubility, ionic charge and size, or molecular mass (based on their interaction with mobile and stationary phases)	High resolving power Compatible with various detectors No need for laborious, time-consuming and chemical-wasting derivatization (required for GC) Can detect non-volatile compounds	Moderate to high cost depending on the system and detectors
GC	Separation based on volatility and interaction with a gas mobile phase and solid stationary phase	Excellent resolving power Transfer analytes from the column to the detector without sample nebulization, enhancing LODs compared to LC Separation of volatile and semi-volatile compounds	Complex sample preparation (derivatization is required)
CE	Separate compounds based on their size, charge and interaction with electric field	High automatization A small volume of injected samples is needed Simple sample preparation	Reduced sensitivity due to the short optical path length and small diameter of the capillary Weak UV absorption Requirement for derivatization Require control of buffer and pH

**Table 4:** Comparison on analytical performance among biosensor methods used for mercury speciation in seafood.

	Sample	Target Analyte	Sample Preparation	Sample Preparation Time	Recognition Element	Signal Transduction	Assay Time	LOD (µg/kg, Method)/(µg/L, Instrumental)	Ref.
1	Fish muscles	MeHg EtHg	Acid extraction with Tris–HNO_3_ buffer	–	DNA-templated Ag–Au alloy nanoparticles	Colorimetric (UV–Vis/naked eye)	~60–70 min	1000 μg/kg	[88]
2	Fish products	iHg	SPE into transparent polymer matrix (TPP) with immobilized Cu (DDTC)_2_	10 min	Hg^2+^-responsive chromogenic reagent	Colorimetric (spectrophotometry/naked eye)	~10 min	8 μg/L	[89]
3	Pig liver and fish	iHg	Acid digestion followed by TSIL extraction	80 min	Task-specific ionic liquid (TSIL) optode	Colorimetric (optical absorbance)	~20 min	0.1 μg/L	[90]
4	Seabass, swordfish	iHg	MAE with HNO_3_ 200 °C	30 min	TPA–TSC fluorescent probe	Fluorescence turn-off	<30 s	<0.04 μg/L	[91]
5	Canned fish	THg	MAE with HNO_3_ and H_2_O_2_	–	Thymine-rich ssDNA on streptavidin-modified MNPs	Fluorescence polarization	–	0.09 μg/L	[92]
6	Fish	MeHg	Alkaline MAE	–	MeHg-specific DNA probe with Ag^0^/Hg^0^ amalgamation	Fluorescence	–	0.089 μg/L	[93]
7	Fish tissue	MeHg EtHg	MAE with HCl	–	DNA extraction by TSIL coupled with sensors for matrix interference	Fluorescence on–off	85 min	1.08 μg/L	[94]
8	Water and fish tissue	MeHg	UAE with HCl	4 min	Carbon-dot nanoprobe	Fluorescence (microfluorospectrometry)		1.18 μg/L	[95]
9	Fish and shrimps	iHg	MAE with HNO_3_	4 h	Poly(ester-urethane) urea–AuNP composite	Electrochemical	<300 s	0.235 μg/L	[96]
10	Seafood products	MeHg	UAE with HCl, pH adjustment	–	MnO_2_/AuNP nanocomposite-modified electrode	Electrochemical	–	0.051 μg/L	[97]
11	Fish muscle	iHg MeHg	UAE with HCl and derivatization	2.5 h	Thymine-rich DNA aptamer	Electrochemical	~60 min	iHg: 0.012 µg/L; MeHg: 0.029 µg/L	[98]

Abbreviations: Triphenylamine–thiosemicarbazone (TPA–TSC); ultrasound-assisted extraction (UAE). LODs are converted from molar concentrations and expressed as μg/L (instrumental LOD) or μg/kg (method LOD) concentrations.

## Data Availability

Data sharing is not applicable. No new data were created or analyzed in this study.

## Supplementary Materials

The following supporting information can be downloaded at: https://www.mdpi.com/article/10.3390/foods15060971/s1, PRISMA 2020 Main Checklist.

## Abbreviations

The following abbreviations are used in this manuscript:

APDC	Ammonia pyrrolidine dithiocarbamate	ID	Isotope dilution
NH_4_Ac	Ammonium acetate	LODs	Limits of detection
AcOH	Acetic acid	LC	Liquid chromatography
AFS	Atomic fluorescence spectrometry	Hg	Mercury
AAS	Atomic absorption spectrometry	MeHg	Methylmercury
CRM	Certified reference material	2-ME	2-Mercaptoethanol
CV-AFS	Cold vapor atomic fluorescence spectrometry	MeOH	Methanol
CVG	Cold vapor generation	MSPE	Magnetic solid phase extraction
CE	Capillary electrophoresis	MNPs	Magnetic nanoparticles
CE–ICP-MS	CE coupled to inductively coupled plasma mass spectrometry	MAE	Microwave-assisted extraction
CNTs	Carbon nanotubes	HNO_3_	Nitric acid
DBD-OES	Dielectric barriers discharge optical emission spectrometry	NPs	Nanoparticles
EtHg	Ethylmercury	PhHg	Phenylmercury
GC	Gas chromatography	RP	Reversed phase
GC-DBD-OES	GC coupled to DBD-OES	SPE	Solid phase extraction
HPLC	High-performance liquid chromatography	THg	Total mercury
HPLC-ICP-MS	HPLC coupled to inductively coupled plasma mass spectrometry	TMAH	Tetramethylammonium hydroxide
HCl	Hydrochloric acid	NaBEt_4_	Sodium tetraethylborate
ICP-MS	Inductively coupled plasma mass spectrometry	NaBPr_4_	Sodium tetrapropylborate
IIP	Ionic imprinted polymer	NaBPh_4_	Sodium tetraphenylborate
IIP-SPE	Ionic imprinted polymer–solid phase extraction	UAE	Ultrasound-assisted extraction
iHg	Inorganic mercury		
